# A Real-Time PCR Approach for Rapid Detection of Viable *Salmonella* Enteritidis in Shell Eggs

**DOI:** 10.3390/microorganisms11040844

**Published:** 2023-03-26

**Authors:** Siew Herng Chan, Sock Hwee Liau, Ying Jia Low, Kern Rei Chng, Yuansheng Wu, Joanne Sheot Harn Chan, Li Kiang Tan

**Affiliations:** 1 National Centre for Food Science, Singapore Food Agency, 7 International Business Park, Singapore 609919, Singapore; 2 Department of Food Science and Technology, National University of Singapore, S14 Level 5 Science Drive 2, Singapore 117542, Singapore

**Keywords:** real-time PCR, rapid detection, viable, *Salmonella* Enteritidis, shell eggs

## Abstract

Rapid and robust detection assays for *Salmonella* Enteritidis (SE) in shell eggs are essential to enable a quick testing turnaround time (TAT) at the earliest checkpoint and to ensure effective food safety control. Real-time polymerase chain reaction (qPCR) assays provide a workaround for the protracted lead times associated with conventional *Salmonella* diagnostic testing. However, DNA-based analysis cannot reliably discriminate between signals from viable and dead bacteria. We developed a strategy based on an SE qPCR assay that can be integrated into system testing to accelerate the detection of viable SE in egg-enriched cultures and verify the yielded SE isolates. The specificity of the assay was evaluated against 89 *Salmonella* strains, and SE was accurately identified in every instance. To define the indicator for a viable bacteria readout, viable or heat-inactivated SE were spiked into shell egg contents to generate post-enriched, artificially contaminated cultures to establish the quantification cycle (Cq) for viable SE. Our study has demonstrated that this technique could potentially be applied to accurately identify viable SE during the screening stage of naturally contaminated shell eggs following enrichment to provide an early alert, and that it consistently identified the serotypes of SE isolates in a shorter time than conventional testing.

## 1. Introduction

*Salmonella* Enteritidis (SE) is one of the most prevalent serovars responsible for foodborne salmonellosis worldwide. Chicken shell eggs play a substantial role as an import vehicle of infections. This invasive bacteria strain can be found in the egg contents before oviposition by SE-infected laying hens or penetrate through from the eggshell surface [1,2]. Reports from the European Union (EU) and European Economic Area (EEA) have identified SE in clinical cases of salmonellosis and confirmed that the consumption of SE-contaminated eggs was a major source for the outbreak [3,4]. In Singapore, SE has also been identified as a serovar frequently associated with foodborne salmonellosis; there were 1152 reported cases between 2012 to 2016, and SE ranked in the top 25% of serovars among the clinical cases [5]. Egg-associated SE presents a significant public health risk given the common culinary practice of using raw shell eggs as ingredients for meals and desserts, as well as the unique dietary habit of consuming them undercooked. Hence, interventions at the earliest possible opportunity via end-product checks are essential as upstream measures for the reduction of potential risk exposure through the consumption of SE-contaminated raw shell eggs.

Robust and accurate testing for viable SE in shell eggs is essential for early alerts and work as an assurance to facilitate the smooth supply of safe raw shell eggs to retail markets. Conventional bacteriological culture-based methods for *Salmonella* detection referenced from the US Food and Drug Administration (FDA) Bacteriological Analytical Manual (BAM) require enrichment, plating on selective media with subsequent biochemical identification, and serotyping based on the Kauffman–White scheme to elucidate the bacterial surface ‘O’ and ‘H’ antigens using specific antisera [6]. While traditional *Salmonella* serotyping by slide agglutination remains the ‘gold standard’, the process often requires several selective culturing steps, numerous panels of antisera, and technical skills and experience, and results may be ambiguous at times [7,8]. A cultured-based system that provided live isolates would typically require five to seven days for SE confirmation via “gold standard” and biochemical testing, hampering the throughput of sample testing [9]. Furthermore, there have been reports of false-positive *Salmonella* spp. detected via biochemical testing of colony isolates on non-*Salmonella* bacteria, such as *Citrobacter* spp. and *Proteus* spp., which share some morphological and biochemical similarities with *Salmonella* [10,11,12].

Rapid molecular-based methodologies, such as real-time polymerase chain reaction (qPCR), are alternatives that can improve the accuracy and efficiency of SE testing and confirmation [13,14,15,16,17]. Unlike the traditional *Salmonella* culture-based approaches, several investigations have shown that qPCR methods provide superior efficiency and specificity in detecting SE in a variety of food matrices, including shell eggs [16,18,19]. Nevertheless, DNA from dead cells may be falsely perceived as a positive readout of viable bacteria and result in an overestimation of the viable cell numbers [20]. These qPCR tests may also be affected by the inhibitory effects of food components, which can lead to false negative results and increase the risk of contracting foodborne illnesses if contaminated food is consumed [21]. Thus, it is critical to accurately detect viable bacteria for regulatory decision making and the management of associated risks.

The aims of this study were to develop an SE qPCR method for the rapid screening of viable SE in shell eggs as well as confirmation of the SE serotype amongst the *Salmonella* colonies in a reduced testing turnaround time. We have demonstrated that the SE qPCR can effectively differentiate between viable and non-viable SE using the difference in Cq values observed in post-enriched egg cultures and provide confirmation of the SE serotype from the *Salmonella* colonies. Our findings have highlighted the potential for the SE qPCR assay to be integrated into the conventional *Salmonella* process to accelerate the detection of SE in shell eggs.

## 2. Materials and Methods

### 2.1. Salmonella Strains

A total of 89 *Salmonella* strains across 6 different serogroups were selected for specificity testing and artificial inoculation studies (Table S1). These strains were plated on tryptic soy agar (TSA) plates (Merck, Rahway, NJ, USA) and incubated overnight at 37 °C for 24 h prior to nucleic acid extraction from colonies or inoculation into a shell egg mixture.

### 2.2. Preparation of Raw Shell Eggs for Enrichment

The process of preparing the shell eggs for *Salmonella* testing was adapted from the US FDA BAM Chapter 5 [6]. The shell eggs were checked for physical defects, and any debris was removed. The eggs were surface sanitized using 70% (*v*/*v*) ethanol and then air dried at room temperature. Each egg was aseptically cracked with a sterile spoon, and the raw egg contents from 20 eggs were pooled into sterile stomacher bags for homogenization in a stomacher for 30 s. A volume of 25 mL of homogenized raw eggs was transferred into a new, sterile stomacher bag containing 225 mL of 1 × buffered peptone water (BPW) (Bio-Rad, Paris, France). The homogenized egg mixture was incubated at 35 °C for 16 h to 24 h.

### 2.3. Artificial Contamination of Live and Heat-Inactivated SE in Shell Eggs

To set up the SE cultures for viable testing in shell eggs, a single SE colony was inoculated into 10 mL of universal pre-enrichment broth (UPB) (Neogen, Birkenhead, UK) in a 50 mL falcon tube and incubated for 5 h to 6 h to achieve an optical density (OD) of 1.0. The incubated SE culture was diluted with UPB to obtain an OD of approximately 0.1, which corresponded to an estimated total live cell concentration of 10^7^ CFU. To obtain non-viable SE, diluted SE culture was heat-inactivated at 121 °C for 15 min. The viability of both the live and heat-inactivated SE was confirmed by plating on TSA agar and incubation at 35 °C for 16 h to 24 h to check for bacteria growth. Two independent studies were conducted. The experimental parameters and *Salmonella* strains used are detailed in Table S2.

To demonstrate the dosage effect, homogenized raw eggs were prepared as described above and spiked with different diluted dosages of live and heat-inactivated SE (Table S2). The inoculated egg cultures were further homogenized and incubated overnight for 16 h at 37 °C. The cell concentrations of live and heat-inactivated SE from the artificially contaminated egg samples were enumerated by standard plate counting on xylose lysine deoxycholate (XLD) agar plates (Thermo Scientific Microbiology, Melaka, Malaysia) after overnight incubation at 37 °C. Inactivated SE was confirmed on a Salmonella-selective agar plate with no growth detected.

### 2.4. Isolation and Identification of Salmonella Enteritidis Using Conventional Workflow in Naturally Contaminated Shell Eggs

Seventy-three naturally contaminated shell eggs were prepared, homogenized, and incubated as mentioned above. After incubation, 10 µL of enriched culture from each egg sample was regrown in brain–heart infusion (BHI) broth (Oxoid, Basingstoke, UK) at 37 °C for 3 h. To prepare the sample lysate, 5 µL of regrown culture was added into 200 µL of BAX^®^ System Lysis Buffer (Hygiena, Camarillo, CA, USA) supplemented with BAX^®^ protease (Hygiena, Camarillo, CA, USA). These sample lysates were heat-treated, first at 37 °C for 20 min and then at 95 °C for 10 min, before being cooled at 4 °C for 5 min. Each sample lysate of 30 µL was added into the *Salmonella* PCR tablets for the BAX^®^ System and loaded into the BAX^®^ Q7 (DuPont Qualicon, Hygiena, Camarillo, CA, USA). Results were obtained after approximately 1.5 h. Samples in which *Salmonella* spp. was not detected were reported as negative. Samples positive for *Salmonella* spp. were concentrated using *Salmonella*-specific immunomagnetic beads (Dynabeads^®^ anti-*Salmonella*, Applied Biosystems™, Vilnius, Lithuania) on an automated immunomagnetic separation (IMS) system (BeadRetriever™, Thermo Fisher Scientific, Vantaa, Finland). Each sample had a 100 µL eluate of magnetic beads, of which 10 µL was streaked onto each XLD, Hektoen enteric (HE) (Oxoid, UK), and chromogenic *Salmonella* (Oxoid, Wesel, Germany) agar plate for *Salmonella* isolation. All agar plates were incubated at 35 °C for 18 h to 24 h prior to screening for presumptive *Salmonella* colonies. Lysine iron agar (LIA) and triple sugar iron (TSI) agar slants were inoculated with presumptive *Salmonella* colonies to screen for the presence of *Salmonella*. Based on colorimetric changes in the LIA (Thermo Scientific Microbiology, Malaysia) and TSI (Thermo Scientific Microbiology, Malaysia), suspected *Salmonella* colonies were tested with slide agglutination using rabbit antiserum for poly “O” (SSDI Diagnostica, Hillerød, Denmark) and MAST^®^ assure antiserum for poly “H” (Mast Group, Bootle, UK) to confirm the presence of *Salmonella*. Upon *Salmonella* confirmation, the colonies were streaked onto XLD agar plates for overnight incubation. Colonies on XLD agar plates were subsequently streaked onto TSA with 5% sheep blood plates (Thermo Scientific Microbiology, Malaysia) for an overnight incubation for downstream biochemical confirmation by Vitek2^®^ (bioMérieux, Inc., Marcy-l’Étoile, France) using Vitek2^®^ GN ID cards (bioMérieux, Inc., France). The colonies were further inoculated on nutrient agar slants (Oxoid, UK) for another overnight incubation to conduct serotyping using the slide agglutination technique (SSDI Diagnostica, Hillerød, Denmark). Slide agglutination was conducted using highly specific agglutinating antisera to identify the presence of the “O” (somatic) and “H” (flagellar) antigens for *Salmonella*. The unique combinations of somatic and flagellar antigens for each isolate were referenced to the Kauffman–White scheme (K-W scheme) to determine the specific *Salmonella* serotype [22].

### 2.5. SE qPCR Assay

#### 2.5.1. Specificity of SE qPCR Assay in Isolate Confirmation

Selected *Salmonella* strains were cultured as described above before nucleic acid extraction from single colonies for isolate confirmation by SE qPCR.

#### 2.5.2. DNA Extraction from Salmonella Colonies and Incubated Shell Egg Cultures

For the *Salmonella* colonies, DNA extraction was also performed using the foodproof^®^ StarPrep Three kit (Hygiena, Marl, Germany). Single colonies were picked from respective TSA or XLD plates and resuspended in 200 µL of lysis buffer from foodproof^®^ StarPrep Three (Hygiena, Germany) for cell lysis to yield a cell lysate for qPCR.

Similarly, DNA was extracted from the artificially contaminated or naturally contaminated shell egg cultures using the foodproof^®^ StarPrep Three kit (Hygiena, Marl, Germany) according to manufacturer’s instructions, but with modifications. Briefly, 50 µL was aliquoted from each incubated egg culture into 1.5 microcentrifuge tubes before centrifugation at 13,000× *g* for 5 min. The supernatant was decanted and 200 µL of lysis buffer from the foodproof^®^ StarPrep Three kit (Hygiena, Marl, Germany) with 5 µL of protease (Hygiena, Marl, Germany) were added. Each egg sample was resuspended by pipetting or vortexing before incubation at 50 °C for 10 min and heating at 95 °C for 10 min in a heating unit for cell lysis. After cell lysis, each sample tube was cooled to room temperature and centrifuged at 13,000× *g* for 5 min. The supernatant contained extracted DNA and could be used directly for qPCR.

#### 2.5.3. Real-Time PCR Assays for SE

Real-time PCR (qPCR) assays were performed using the foodproof^®^ *Salmonella* Genus plus Enteritidis and Typhimurium Detection Lyokit (Hygiena, Marl, Germany) to detect SE, as per the manufacturer’s protocol. Each target was represented by a unique reporter dye, i.e., FAM, HEX, and ROX for *Salmonella* Enteritidis, *Salmonella* Typhimurium, and *Salmonella* spp., respectively. Briefly, 25 µL of cell lysate was transferred into each PCR tube to reconstitute the lyophilized reagents. All the DNA samples were tested and analyzed in a Quantstudio™ 5 real-time thermocycler (Applied Biosystems, Waltham, MA, USA). Cycling conditions consisted of an initial incubation at 37 °C for 4 min followed by an initial denaturation at 95 °C for 5 min, and, subsequently, 50 cycles of 5 s at 95 °C and 60 s at 60 °C. No-template controls (NTC) and positive controls, consisting of 25 μL of PCR-grade water and 25 μL of positive DNA template, respectively, were included for each qPCR assay.

### 2.6. Data Analysis

The real-time PCR results for SE were analyzed using QuantStudio™ Design and Analysis, provided in the Quantstudio™ 5 real-time thermocycler (Applied Biosystems, USA). Each SE qPCR assay included 1 non-template control (NTC) and 1 positive control. Samples with Cq numbers higher than 40 were considered not to have detected the specific target.

## 3. Results

We have developed an SE qPCR approach to expedite the detection of SE during the screening of shell eggs and reduce the lengthy testing turnaround time needed to verify the serotype of SE isolates. The SE qPCR assays proved to be highly specific when performed on well-characterized reference strains and wild type SE isolates. The Cq values of the SE qPCR test, which were generated from viable and heat-inactivated post-enriched egg cultures artificially contaminated with SE, could be used to identify the viable SE from the post-enriched egg cultures of naturally contaminated eggs. We demonstrated that the assay is comparable to the “gold standard” when verifying the identity of SE isolates acquired from naturally contaminated eggs.

### 3.1. Specificity of SE qPCR Assay

A total of 89 *Salmonella* strains were analyzed using the SE qPCR assay (Table S1). Out of these 89 *Salmonella* strains, 15 out of 15 SE strains were confirmed as SE positive, and the remaining 74 non-SE strains were confirmed as SE negative. The latter group included 10 non-Enteritidis belonging to serogroup D, which frequently results in false-positive results when using other DNA kits with less specificity due to cross-reactivity issues (Table 1). The inclusivity test demonstrated that the SE qPCR assay is specific for SE detection with no cross-reactivity with other non-Enteritidis serotypes in serogroup D or other serogroups.

### 3.2. Detection of SE in Artificially Contaminated Egg Samples Using SE qPCR Assay

Two independent studies were performed to determine whether the change in Cq could be used to differentiate between live and heat-inactivated SE in artificially contaminated egg samples. Accordingly, two strains of SE were cultured and prepared to obtain varying concentrations of live and heat-inactivated SE for inoculation into egg samples for overnight incubation (Table 2). For the 0 h post-inoculated (hpi) egg samples, no SE was detected for any of the samples, except the sample inoculated with 10^6^ CFU/25 g of heat-inactivated SE. The samples spiked with three different concentrations of live SE and 10^6^ CFU/25 g of heat-inactivated SE were collected at 16 hpi and tested positive for SE using the SE qPCR (Table 2). However, only the samples spiked with live SE had a change in Cq from “Not Detected” to a mean range of 17.4 to 17.8, regardless of the initial SE concentration. The Cq values for all 16 hpi samples with 10^6^ CFU/25 g of heat-inactivated SE remained above 35.

Eight strains of SE were cultured and inoculated in raw eggs to obtain a live SE concentration of <10 CFU/25 g for 16 h of incubation. Similarly, no SE was detected with the SE qPCR in any of the 0 hpi egg samples (Table 3). However, changes in Cq values ranging from 16.1 to 25.9 were observed for all 16 hpi samples (Table 3). Based on the above findings, we observed that a Cq range of approximately 16.0 to 26.0 was indicative of the presence of viable SE, while a Cq range of approximately 35.0 to 40.5 represented the presence of non-viable SE. No sample was detected with a Cq range between 26.0 and 35.0.

### 3.3. Comparison of Selectivity Performance of SE qPCR Detection Method vs. Conventional Salmonella qPCR Method in Naturally Contaminated Shell Eggs

A total of 73 naturally contaminated shell eggs were tested using the SE qPCR assay concurrently with the conventional *Salmonella* workflow (Figure S1). We observed that when screening using the *Salmonella* qPCR assay, six samples were determined to be *Salmonella* spp. positive. Using the Cq range established in the artificially contaminated eggs, the SE qPCR assay identified three samples (Cq ≤ 25) as indicative of viable SE, one sample as likely for non-viable SE (Cq = 34.0), and two other samples as *Salmonella* spp. positive through the screening testing at day 2 (Table 4). Isolates from Samples 1 to 3 and Samples 5 to 6 were confirmed as SE (Cq ≤ 25) and non-SE, respectively, using SE qPCR at day 4. As no isolate was cultured from Sample 4, the SE detected in the post-enriched egg culture during screening was confirmed as non-viable (Table 4). The results from the agglutination-based serotyping corroborated the results from the SE qPCR, though they required an additional three days to obtain. Taken together, we suggest that Cq ≤ 30 be used as an indicator of viable SE during screening and isolate confirmation.

## 4. Discussion

Our findings have demonstrated that the rapid SE qPCR assay can be integrated into a standard food testing method which can detect viable SE by day 2 and confirm the SE serotype in isolates by day 4, a noticeably shortened testing turnaround time compared with the conventional methodology. As qPCR is a highly sensitive method, there is a risk of false-positive PCR signals due to the amplification of nucleic acids originating from dead cells [23]. Hence, subculturing from viable SE positive samples detected on day 2 is required to obtain pure colonies for SE confirmation on day 4. Viability differentiation in post-enriched egg cultures using Cq values as an indicator has been previously documented, primarily for estimating *Salmonella* concentration where Cq values over 34.0 were observed when high doses of non-viable *Salmonella* (10^6^ CFU/mL) were spiked into egg cultures [24]. Here, we demonstrated the effectiveness of utilizing our established Cq values in naturally infected shell eggs to improve the detection approach and enable quick reporting as well as early interventions to stifle this important vehicle of disease.

SE is the most prevalent serotype in clinical infections, and shell eggs are implicated as a source. Additionally, *Salmonella* spp. is one of the most prevalent pathogens with a high incidence of antibiotic-resistant (AMR) genes, and this is particularly critical for public health [25]. This includes the emergence of multi-drug resistant (MDR) bacteria, such as SE associated with poultry products, which are potential sources for the spread of MDR SE [26,27,28]. The increasing prevalence of MDR *Salmonella* results in an elevated health risk through the increased likelihood of acquiring infections by cross-contamination during food handling or the consumption of contaminated food [29]. The emergence of MDR pathogens in food sources necessitates the development of rapid diagnostic tools such as specific qPCR assays to screen and monitor for foodborne pathogens and ensure early detection [30,31,32]. Ultimately, a combination of rapid diagnostic tools and cultural methods will provide improved efficiency and confidence in the detection of pathogens, which is essential to prevent the importation or sale of food contaminated with unsafe levels of pathogenic microorganisms [33].

Molecular tests to serotype *Salmonella*, such as whole genome sequencing and microarrays, are alternatives which circumvent the lengthy testing turnaround time and reduce uncertainty [7,34,35,36]. WGS can enhance discriminatory data for *Salmonella* isolates, which can be used to accurately determine *Salmonella* serotypes [37,38]. WGS provides comprehensive strain characterization for epidemiology research with high resolution and the ability to distinguish between closely related isolates. However, the workflow of WGS requires infrastructure, bioinformatics knowledge, and a comparatively longer duration to generate data. Additionally, WGS is also limited by the need for a pure isolate, which will not be available during the screening of post-enriched egg cultures [39]. These limitations, compared with those of other molecular methods such as qPCR, makes WGS less attractive and effective as a diagnostic approach for high throughput [39]. Several studies have shown the benefits of qPCR for SE detection, including the possibility of multiplexing to identify different *Salmonella* serovars and its specificity for SE based on specific gene markers [35,40,41]. Here, we have demonstrated that the SE qPCR assay is highly specific to SE DNA extracted from the SE colonies and did not display any cross-reactivity with other non-SE strains and thereby cause false-positives (Table 1). Our evaluations of other commercially available qPCR assays revealed cross-reactions to specific serogroup D isolates (data not shown). Adaption of this SE qPCR assay for SE serotype confirmation from presumptive *Salmonella* colonies can reduce the need for further biochemical testing and agglutination-based serotyping. This can significantly shorten the SE testing turnround time and boost the sample throughput. While the main emphasis of this study was SE detection, this multiplex qPCR assay can also detect *Salmonella* Typhimurium. Further investigations are required to determine whether *Salmonella* Typhimurium cross-reactivity problems exist.

Other rapid *Salmonella* quantification methods based on qPCR have also been developed for the absolute quantification of *Salmonella* without overnight enrichment to eliminate the overall testing turnaround time. For example, the TyphiTyper LAMP assay is a rapid and simple test that can accurately identify typhoidal *Salmonella* serovars and discriminate between *Salmonella* species [42]. The detection of SE using LAMP assays has been effectively demonstrated in liquid eggs (liquid egg components are potentially inhibitory in PCR assays if untreated) [43]. Although LAMP assays can be more sensitive and cheaper to perform than qPCR assays, there may be an increased likelihood of false-positives, and sequencing of the amplicons may be needed for confirmation, which requires additional labor and time [43]. Additionally, most of these assays often fail to differentiate between the DNA detected from viable and non-viable cells. The detection of viable SE is essential because viable cells can cause diseases such as salmonellosis if consumed. Other methods, such as the pre-treatment of samples with photoreactive binding dyes (i.e., propidium monoazide) to differentiate the viability of bacterial cells, including *Salmonella* spp. and SE, prior to qPCR or LAMP, have also been reported [44,45,46,47]. These photoreactive binding dyes can penetrate the compromised cell membranes of dead or injured cells, bind irreversibly to cellular DNA, and prevent the amplification of the target DNA during qPCR [48]. However, this treatment requires additional time for photoactivation, and the complexity of the sample matrix may reduce the efficiency with which the dye suppresses dead cell signals, leading to an overestimation of the number of intact cells [49]. It has also been suggested that these photoreactive binding dyes can potentially reduce the signals of viable bacterial cells at low concentrations, resulting in false-negatives, which are unacceptable for SE due to the zero-tolerance stance adopted for this pathogen [49].

Here, we developed a rapid and robust methodology based on a qPCR assay with a Cq value cut-off which can indicate the presence of viable SE during the screening of egg cultures as well as expedite the confirmation of SE serotype based on isolates.

## 5. Conclusions

The SE qPCR assay tested in this work can be employed as a quick detection technique to deliver strong suggestive indications of the presences of viable SE in post-enriched egg cultures by the second day. Additionally, it can be utilized as a downstream serotyping confirmation tool to serotype *Salmonella* colonies without the requirement for time-consuming steps for slide agglutination serotyping. Overall, we have shown that the application of the SE qPCR in the current testing system is simple and enables the rapid identification of viable and contaminating *Salmonella* serotypes, facilitating earlier regulatory intervention to strengthen the regulation of food safety control and management.

## Figures and Tables

**Table 1:** Evaluation of the specificity of SE qPCR assay.

Serogroup	Serotype	Isolates (n)	SE +/−
B	−	23	−
C	−	22	−
D	Enteritidis	15	+
	Non-Enteritidis	10	−
E	−	10	−
G	−	4	−
I	−	5	−
	Total	89	

**Table 2:** Changes in Cq values for detection of live and heat-inactivated SE in 16 h post-inoculated artificially contaminated egg samples.

*Salmonella* Serotype	Post-Inoculation (h)	SE qPCR, Cq				
		Live SE (CFU/25 g)			Heat-Inactivated SE (CFU/25 g)	
		<10	10	10^1^	10^3^	10^6^
Enteritidis ATCC 13076	0	N.D ^a^	N.D	N.D	N.D	+(37.7)
	16	+ ^b^ (17.4) ^c^	+(17.8)	+(17.7)	N.D	+(40.4)
						
Enteritidis 215/20	0	N.D	N.D	N.D	N.D	+(35.3)
	16	+(17.4)	+(17.6)	+(17.7)	N.D	+(36.7)

^a^—SE not detected; ^b^—SE positive; ^c^—Cq values; values in parentheses are means of duplicates at a fixed threshold.

**Table 3:** Detection of live SE in artificially contaminated egg samples.

*Salmonella* Serotype	Post-Inoculation (h)	SE qPCR, Cq	*Salmonella* Serotype	Post-Inoculation (h)	SE qPCR, Cq
		Live SE < 10 CFU/25 g			Live SE < 10 CFU/25 g
Enteritidis 050/19	0	N.D ^a^	Enteritidis 111/19	0	N.D
	16	+ ^b^ (17.7) ^c^		16	+(16.1)
Enteritidis 062/18	0	N.D	Enteritidis 155/20	0	N.D
	16	+(18.2)		16	+(20.5)
Enteritidis 063/21	0	N.D	Enteritidis 346/18	0	N.D
	16	+(24.4)		16	+(25.9)
Enteritidis 079/20	0	N.D	Enteritidis 360/19	0	N.D
	16	+(19.9)		16	+(24.8)

^a^—SE not detected; ^b^—SE positive; ^c^—Cq values; values in parentheses are means of duplicates at a fixed threshold.

**Table 4:** Comparison of SE detection in naturally contaminated eggs using SE qPCR and conventional workflow for *Salmonella*.

Sample	SE qPCR						Conventional Workflow		
	Screening (Day 2)			Isolate Confirmation (Day 4)			Screening (Day 2)	Isolate Confirmation ^a^ (Day 7)	
	Viable SE	Non-Viable SE	*Sal*. spp.	SE	Non-SE		*Sal*. spp.	SE	Non-SE
1	+(18.1) ^b^		+	+(16.0)			+	+	
2	+(18.3)		+	+(17.7)			+	+	
3	+(25.0)		+	+(15.5)			+	+	
4		+(34.0)	+	No isolate			+	No isolate	
5			+			+	+		+
6			+			+	+		+

^a^—Serotyping by slide agglutination technique; ^b^—Cq values, in parentheses.

## Data Availability

The data presented in this study are available on request from the corresponding author.

## Supplementary Materials

The following supporting information can be downloaded at: https://www.mdpi.com/article/10.3390/microorganisms11040844/s1, Figure S1: Workflows for detection of *Salmonella* Enteritidis in shell eggs; Table S1: *Salmonella* strains for inclusivity and artificial contamination of shell eggs; Table S2: Experimental parameters and *Salmonella* Enteritidis strains used for artificial contamination of shell eggs.
