# Supplementary material for: A Real-Time PCR Approach for Rapid Detection of Viable Salmonella Enteritidis in Shell Eggs
Source: Microorganisms. 2023 Mar 26;11(4):844. doi: 10.3390/microorganisms11040844 (PMC10143610; doi:10.3390/microorganisms11040844)
Supplement: Supplementary file 1 [file microorganisms-11-00844-s001.zip › microorganisms-2172827-supplementary.pdf]

Supplementary Materials

Table S1: *Salmonella* strains for inclusivity and artificial contamination of shell eggs

Salmonella serotype and strain designation	Source of isolate	Year of isolate	Inclusivity[a] / Artificial contamination[b]	*Salmonella* serotype and strain designation	Year of isolate	Inclusivity[a] / Artificial contamination[b]
Enteritidis ATCC 13076	ATCC	N.A	a,b	Corvallis 391/19	2019	a
Enteritidis ATCC 4931	ATCC	N.A	a,b	Derby 365/19	2019	a
Enteritidis ATCC 31194	ATCC	N.A	a,b	Derby 030/20	2020	a
Javiana ATCC 10721	ATCC	N.A	a	Derby 201/20	2020	a
Enteritidis 062/18	Lab iso-	2018	a,b	Derby 191/21	2021	a
Enteritidis 346/18	lates from	2018	a,b	Dublin 101/09	2009	a
Enteritidis 356/18	poultry,	2018	a,b	Dublin 358/17	2017	a
Enteritidis 050/19	bovine,	2019	a,b	Dublin 385/17	2017	a
Enteritidis 111/19	crocodile,	2019	a,b	Dublin 570/17	2017	a
Enteritidis 360/19	seafood,	2019	a,b	Give 003/15	2015	a
Enteritidis 44/20	ready-to-	2019	a,b	Give 426/15	2015	a
Enteritidis 53/20	eat foods	2020	a,b	Give 253/18	2018	a
Enteritidis 079/20	and envi-	2020	a,b	Give 254/18	2018	a
Enteritidis 150/20	ronmental	2020	a,b	Give 049/20	2020	a
Enteritidis 155/20	swabs	2020	a,b	Give 162/20	2020	a
Enteritidis 215/20		2020	a,b	Hadar 021/10	2010	a
Agona 209/21		2021	a	Hadar 709/11	2011	a
Berta 72/20		2020	a	Hadar 436/13	2013	a
Berta 73/20		2020	a	Hadar 050/14	2014	a
Braenderup 158/17		2017	a	Hadar 252/16	2016	a
Braenderup 210/17		2017	a	Havana 588/07	2007	a
Brancaster 349/18		2018	a	Havana 181/11	2011	a
Corvallis 161/17		2017	a	Havana 562/11	2011	a
Corvallis 197/19		2019	a	Havana 055/16	2016	a

Salmonella serotype and strain designation	Year of isolate	Inclusivity[a] / Artificial contamination[b]	*Salmonella* serotype and strain designation	Year of isolate	Inclusivity[a] / Artificial contamination[b]

Heidelberg 155/18	2018	a	Schwarzengrund 194/21	2021	a
Heidelberg 164/20	2020	a	Stanley 128/21	2021	a
Heidelberg 177/21	2021	a	Thompson 029/16	2016	a
Heidelberg 207/21	2021	a	Typhimurium 023/20	2020	a
Heidelberg 211/21	2021	a	Typhimurium 034/20	2020	a
Houston S284/19	2019	a	Typhimurium 120/20	2020	a
Hvittingfoss 182/14	2014	a	Typhimurium 142/21	2021	a
Hvittingfoss 216/14	2014	a	Typhimurium 183/21	2021	a
Hvittingfoss 233/15	2015	a	Typhimurium 208/21	2021	a
Hvittingfoss 266/15	2015	a	Typhimurium 210/21	2021	a
Hvittingfoss 232/16	2016	a	Virchow 485/18	2018	a
Infantis 274/18	2018	a			
Infantis 188/19	2019	a			
Infantis 200/19	2019	a			
Infantis 292/19	2019	a			
Infantis 388/19	2019	a			
Java 062/11	2011	a			
Javiana S365/18	2018	a			
Kentucky 104/15	2015	a			
Kentucky 236/15	2015	a			
Kentucky 063/16	2016	a			
Kentucky 252/17	2017	a			
Liverpool 248/17	2018	a			
Liverpool 221/18	2018	a			
Liverpool 373/18	2021	a			
St. Paul 61/18	2021	a			
St. Paul 179/21	2021	a			

Table S2: Experimental parameters and *Salmonella* Enteritidis strains used for artificial contamination of shell eggs

Study No.	*Salmonella* serotype and strain designation	*N*	Final concentration of live SE (CFU/25 mL)	Final concentration of heat-killed SE (CFU/25 mL)	Incubation time (h)
1	Enteritidis ATCC 13076<br>Enteritidis 215/20[i]	2	<10, 10, 100	10^3, 10^6	0, 16, 24
2	Enteritidis 050/19[i]<br>Enteritidis 062/18[i]<br>Enteritidis 063/21[i]<br>Enteritidis 079/20[i]<br>Enteritidis 111/20[i]<br>Enteritidis 155/20[i]<br>Enteritidis 215/20[i]<br>Enteritidis 346/18[i]<br>Enteritidis 360/19[i]	9	<10	N.A	0, 16, 24

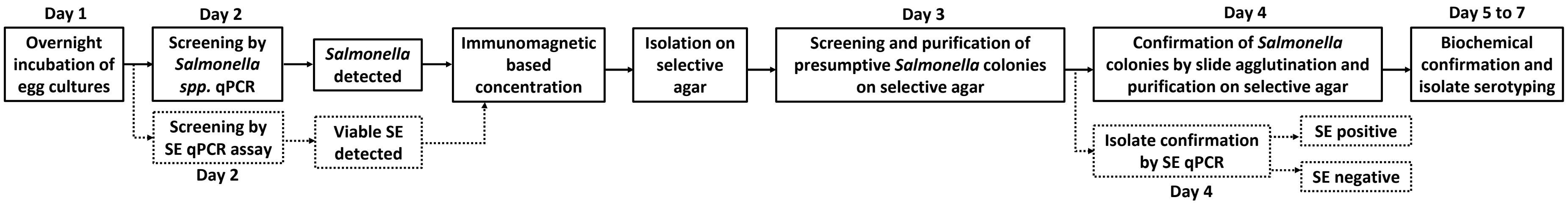

Figure S1: Workflows for detection of *Salmonella* Enteritidis in shell eggs. For conventional cell-culturing methodology for *Salmonella* spp. (solid line), it requires up to additional 5 days to determine the *Salmonella* serotype if *Salmonella* was detected during screening of incubated egg cultures. In contrast, application of SE qPCR assay (dashed line) for screening provides early insights on the presence of viable SE. Additionally, SE qPCR assay (dashed line) can rapidly confirm SE serotype in isolates as compared to biochemical testing and agglutination-based serotyping.
